# Chitosan Hydrogels for Antibiotic Remediation and Dye Removal: A Review

**DOI:** 10.3390/gels12080658

**Published:** 2026-07-23

**Authors:** Sai Yin, Wen Yuan, Longmei Zhao, Yida Niu, Jianhui Guo

**Affiliations:** 1 College of Fine Arts, Henan University, Kaifeng 475001, China; bigyinsai@outlook.com (S.Y.); 18736501854@163.com (W.Y.); 2 Jinshui Branch of Zhengzhou Natural Resources and Planning Bureau, Zhengzhou 450002, China; 18838285182@163.com; 3 College of Art, Zhengzhou University of Science and Technology, Zhengzhou 450064, China; niuyida@zit.edu.cn

**Keywords:** chitosan, hydrogels, molecular modification, antibiotic remediation, dye removal

## Abstract

The co-contamination of aquatic environments by antibiotic residues and organic dyes poses a serious threat to ecological security and human health, underscoring the urgent need for high-efficiency, recyclable, and environmentally benign adsorbents. Chitosan, a naturally occurring alkaline polysaccharide rich in reactive functional groups, has attracted considerable attention in water treatment applications. Nevertheless, its practical use is often constrained by intrinsic limitations, including poor stability in acidic media, inadequate mechanical strength, and difficulties in solid–liquid separation. Chitosan-based hydrogels, featuring unique three-dimensional cross-linked networks, high porosity, and strong hydrophilicity, provide efficient mass-transfer pathways for macromolecular contaminants and thus offer a promising strategy to overcome the shortcomings of pristine chitosan. This review comprehensively summarizes recent advances in chitosan-based hydrogel adsorbents, with a focus on elucidating the critical structure–performance relationships that link molecular/structural design to adsorption efficacy. First, fabrication strategies are systematically reviewed, ranging from molecular-level modifications (e.g., grafting, chemical cross-linking, and interpenetrating polymer networks) to macroscopic structural engineering approaches (e.g., mechanically reinforced, magnetic, and stimuli-responsive hydrogels). Subsequently, adsorption behaviors toward representative classes of antibiotics, including tetracyclines, fluoroquinolones, and sulfonamides, are critically examined, with emphasis on the underlying mechanisms such as electrostatic interactions, hydrogen bonding, π–π stacking, and pore-filling effects. In addition, the removal performance of chitosan-based hydrogels for organic dyes with varying charge characteristics is summarized, together with an analysis of how environmental factors (e.g., pH and ionic strength) influence adsorption kinetics and thermodynamics. Finally, key challenges related to mechanical robustness, selective adsorption, and recyclability are discussed, and future perspectives are proposed for the development of multifunctional, synergistic, and intelligent, environmentally responsive chitosan-based hydrogel materials. This review aims to provide systematic insights and guidance for the rational design of advanced hydrogel adsorbents for the treatment of complex wastewater.

## 1. Introduction

Driven by rapid global industrialization, the expansion of industries such as pharmaceutical manufacturing, intensive livestock farming, and textile dyeing has resulted in massive discharges of antibiotics and organic dyes into natural water bodies, giving rise to persistent and complex environmental contamination challenges [1,2,3,4]. To fully grasp the scope of this issue, it is essential to understand the primary classes of these pollutants. The antibiotics most frequently detected in aquatic environments generally belong to four major classes: tetracyclines (e.g., tetracycline, oxytetracycline), fluoroquinolones (e.g., ciprofloxacin), sulfonamides (e.g., sulfamethoxazole), and β-lactams (e.g., amoxicillin). These emerging contaminants exhibit a distinctive “pseudo-persistence”: even at trace concentrations, they can strongly promote the emergence and dissemination of antibiotic resistance genes among aquatic microorganisms [5,6]. This process not only disrupts microbial community structure and ecosystem stability but also poses serious risks to human health through biomagnification along the food chain [7]. Meanwhile, organic dyes—broadly classified by their charge and chromophore structures into cationic/basic dyes (e.g., methylene blue, crystal violet), anionic/acidic dyes (e.g., Congo red, methyl orange), and reactive dyes—significantly impair light penetration and reduce dissolved oxygen levels, thereby suppressing the self-purification adsorption capacity of aquatic systems. Moreover, their degradation byproducts, such as aromatic amines, have attracted increasing concern due to their confirmed carcinogenic, teratogenic, and mutagenic effects [8,9]. Given that conventional biological treatments are often inhibited by the bacteriostatic effects of antibiotics, while advanced oxidation processes are typically energy-intensive and costly, the development of broad-spectrum, high-efficiency, and economically viable remediation technologies has become a critical priority in environmental governance.

Among the diverse water remediation strategies, adsorption stands out as one of the most practical and scalable approaches due to its operational flexibility, cost-effectiveness, and minimal risk of secondary pollution [10,11,12]. In particular, motivated by global carbon neutrality targets and green chemistry principles, increasing attention has been devoted to renewable, biomass-derived adsorbents [13,14,15]. Chitosan, derived from the deacetylation of chitin, the second most abundant natural biopolymer on Earth, primarily sourced from crustacean shells, has emerged as a highly promising candidate. As the only naturally occurring cationic, alkaline polysaccharide, Chitosan possesses unique features that make it attractive for environmental remediation. Its polymer backbone is densely populated with highly reactive amino (-NH_2_) and primary/secondary hydroxyl (-OH) functional groups [16,17,18]. These groups not only provide exceptional chelating capabilities and strong electrostatic attraction toward anionic pollutants (driven by the protonation of -NH_2_ groups in slightly acidic to neutral media) but also serve as highly accessible active sites for versatile chemical modifications. Despite these advantages, the direct application of pristine chitosan powder is severely constrained by several engineering limitations, including instability and dissolution under acidic conditions, limited accessibility of active sites due to its low specific surface area, and difficulties in solid–liquid separation arising from its poor mechanical strength.

To overcome these drawbacks, the construction of chitosan-based hydrogels has emerged as an effective and robust strategy [19,20,21]. These materials are characterized by three-dimensional cross-linked networks with interconnected porous architectures that facilitate rapid mass transfer and efficient diffusion of contaminants. Simultaneously, the cross-linked framework markedly enhances physicochemical stability under harsh aqueous environments [22,23,24]. Recent research efforts have focused on improving the adsorption performance of chitosan-based hydrogels along two principal dimensions. At the molecular level, chemical modifications, such as grafting aromatic moieties (e.g., cyclodextrin derivatives) or introducing hydrophobic ligands, are employed to regulate surface polarity and electron density, thereby enhancing selective capture of antibiotics and dyes through mechanisms, including π–π interactions and hydrogen bonding [25,26,27]. At the macroscopic structural level, multi-dimensional architectural designs, such as interpenetrating polymer networks and nanocomposite strategies incorporating carbon nanotubes, metal–organic frameworks, or other functional fillers, are utilized to construct hydrogel frameworks with high specific surface area and robust mechanical integrity, achieving synergistic improvements in adsorption capacity and regenerability [28,29,30].

Although several review articles have previously summarized the application of chitosan hydrogels in water treatment, the rapid evolution of materials science necessitates an updated, more specialized perspective. Most existing reviews either focus broadly on general pollutant removal, treat antibiotics and dyes in isolated contexts, or merely catalog maximum adsorption capacities without critical mechanistic depth. Consequently, a significant knowledge gap persists regarding the synergistic effects of combining molecular-level functionalization with macroscopic structural engineering. Furthermore, recent breakthroughs in advanced nanocomposites (e.g., integration with MXenes or covalent organic frameworks) and the transition from ideal single-solute models to complex, competitive multi-pollutant aquatic environments have not yet been comprehensively synthesized and critically evaluated.

To address these gaps and advance beyond the existing literature, this systematic review provides a comprehensive and critical evaluation of the latest research landscape (with a particular focus on advancements from the past five years) on chitosan-based hydrogels for environmental remediation. Unlike previous reviews, this article places a particular emphasis on the intricate structure–performance relationships, bridging the evolution of fabrication strategies and their direct influence on adsorption performance. The review first outlines the progression of preparation methodologies, from conventional physical and chemical cross-linking to advanced functional modifications and cutting-edge nanocomposite designs. It then critically examines the adsorption behavior of chitosan-based hydrogels toward representative classes of antibiotics and organic dyes, offering in-depth insights into the synergistic intrinsic mechanisms, including electrostatic interactions, pore-size sieving, and coordination complexation. Finally, moving beyond standard laboratory-scale assessments, the remaining challenges associated with selective adsorption in real-world competitive multi-solute systems, mechanical robustness, and engineering scalability are discussed, followed by forward-looking perspectives on the development of next-generation smart, stimuli-responsive, biomass-derived hydrogel adsorbents.

## 2. Fabrication and Construction Strategies of Chitosan-Based Hydrogels

The fabrication and architectural strategies for chitosan-based hydrogels are fundamentally designed to surmount the inherent limitations of natural chitosan, specifically its insufficient mechanical strength, instability in acidic environments, and lack of adsorption selectivity. By precisely modulating cross-linking mechanisms, an optimal balance between pore architecture and mechanical stability can be achieved; notably, chemical cross-linking and graft copolymerization provide the requisite robust backbone support [31,32,33]. In parallel, molecular-level functional modifications are tailored to specific contaminant profiles, incorporating targeted functional groups to enhance the affinity and selectivity of active adsorption sites. Furthermore, in multi-dimensional structural design, implementing strategies such as Interpenetrating Polymer Networks, nanocomposites, and magnetic functionalization enables the material to overcome the constraints of single-component systems in terms of mechanical integrity, adsorption capacity, and solid–liquid separation efficiency. Collectively, these multi-scale construction strategies significantly augment the comprehensive performance and robustness of chitosan-based hydrogels in complex wastewater treatment scenarios.

### 2.1. Cross-Linking Mechanisms of Chitosan Hydrogels

**Figure 1:**
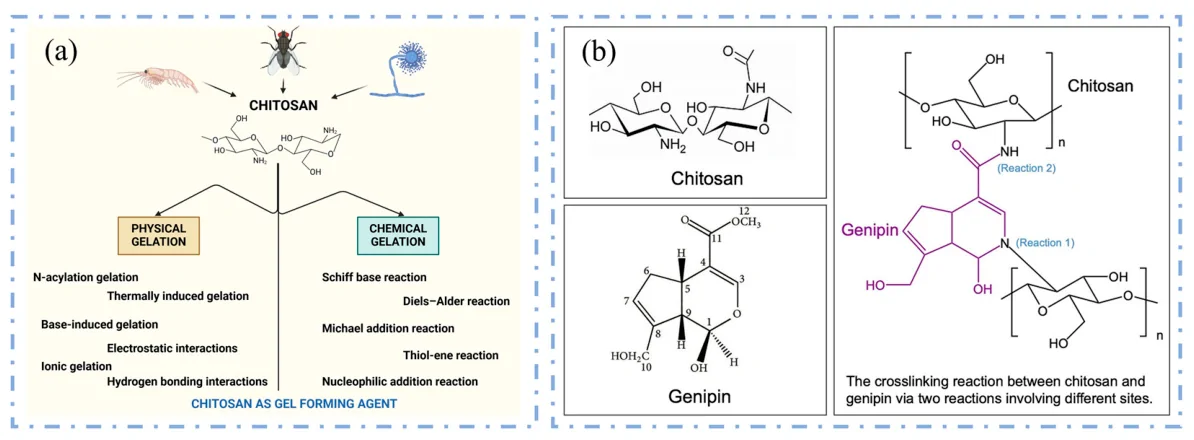
Schematic illustration of various cross-linking strategies and synthesis mechanisms for chitosan-based composite hydrogels. (**a**) Formation mechanism of chitosan gels. Reproduced with permission from Ref. [34]. Copyright 2025, MDPI. (**b**) Covalent cross-linking reaction pathway between chitosan and the natural cross-linker genipin, showing the formation of stable heterocyclic networks. Reproduced with permission from Ref. [37]. Copyright 2025, Elsevier.

The gelation mechanisms of chitosan hydrogels are primarily categorized into physical and chemical cross-linking. Physical cross-linking strategies are characterized by mild reaction conditions and predominantly rely on electrostatic interactions or hydrogen bonding to assemble three-dimensional networks (Figure 1a) [34]. Exemplified by ionic cross-linking with sodium tripolyphosphate, this approach offers facile operation and imparts pH-responsiveness to the material; however, the resulting physical network is relatively loose and susceptible to structural disintegration in high-salinity or extreme pH environments [35]. Analogously, while the freeze–thaw method facilitates the construction of macroporous structures conducive to mass transport, the mechanical strength of the resulting materials often remains limited [36]. In contrast, chemical cross-linking significantly enhances the structural stability and chemical resistance of hydrogels by introducing robust covalent bonds. To prioritize environmental safety, low-toxicity or non-toxic natural cross-linkers (e.g., genipin) are increasingly replacing traditional agents such as glutaraldehyde (Figure 1b) [37]. Furthermore, graft copolymerization strategies, assisted by crosslinking agents, involve the incorporation of monomers, such as acrylic acid, to introduce abundant functional groups while simultaneously forming the cross-linked network. This approach not only enhances the rigidity of the hydrogel backbone but also effectively increases the availability of active adsorption sites [38].

### 2.2. Functional Modification at the Molecular Level

Although natural chitosan possesses abundant amino and hydroxyl groups, the inherent adsorption capacity and selectivity of these functional moieties are often insufficient when treating wastewater with complex matrices. Consequently, targeted chemical modification tailored to the molecular characteristics of specific contaminants is of paramount importance. For cationic dyes and metal ions, the introduction of carboxyl (-COOH) and sulfonic acid (-SO_3_H) groups via grafting reactions significantly potentiates the hydrogel’s electrostatic capture capabilities and ion-exchange performance [39]. Meanwhile, the incorporation of nitrogen- or sulfur-containing moieties, such as thiourea and hydrazone groups, reinforces the coordination affinity for specific heavy metal ions, in accordance with the Hard and Soft Acids and Bases theory [40]. Furthermore, to mitigate the intrinsic inefficiency of the hydrophilic chitosan backbone in removing hydrophobic organic contaminants (e.g., antibiotics and phenols), introducing benzene rings or long-chain alkyl groups has emerged as an effective modification strategy. The former leverages π-π stacking interactions to construct electron-rich regions that efficiently anchor aromatic compounds [41], whereas the latter promotes the formation of hydrophobic microdomains within the hydrogel matrix, facilitating the effective adsorption of non-polar molecules through hydrophobic interactions [42].

### 2.3. Multi-Dimensional Structural Design

Single-component chitosan hydrogels are often constrained by inherent limitations, including insufficient mechanical strength, substantial mass-transfer resistance, and difficulties in recovery. To address these challenges, multi-dimensional structural regulation strategies have been extensively employed to fabricate high-performance composite materials. Regarding mechanical reinforcement, interpenetrating polymer network technology leverages physical entanglements between chitosan chains and polymers such as polyvinyl alcohol (PVA) or polyacrylamide. Mediated by topological interlocking effects, this approach significantly improves the material’s anti-swelling properties and wet strength [43]. To further maximize adsorption capacity, nanofillers such as graphene oxide (GO) [44] and metal–organic frameworks [45] have been incorporated into the hydrogel matrix. These fillers capitalize on their high specific surface areas to optimize the pore structure and induce synergistic adsorption mechanisms. Furthermore, magnetic functionalization provides an efficient solution to the challenge of rapid recovery in engineering applications. By embedding Fe_3_O_4_ nanoparticles via in situ synthesis or blending, the material is endowed with superparamagnetism, enabling rapid separation under an external magnetic field. Moreover, the implementation of core–shell structural designs effectively ensures the chemical stability of the magnetic components within acidic wastewater environments [46].

## 3. Application of Chitosan-Based Hydrogels in Antibiotic Remediation

Chitosan-based hydrogels have demonstrated remarkable adsorption performance for antibiotic remediation, particularly for targeting the removal of diverse antibiotic classes. Regarding tetracyclines, incorporating functional moieties, such as carboxyl and sulfonic acid groups, which into the hydrogels enhances electrostatic interactions and ion-exchange with the antibiotic molecules, thereby significantly elevating adsorption efficacy. In the case of quinolones, structural and functional modifications, such as hybridization with graphene oxide, have been employed to optimize adsorption mechanisms, thereby markedly boosting the removal efficiency of these drugs. Furthermore, regarding sulfonamides and other antibiotics, the introduction of nitrogen- or sulfur-containing groups alongside hydrophobic modifications has enhanced the material’s affinity and selectivity, enabling efficient sequestration of a broad spectrum of antibiotics. By integrating these attributes, chitosan-based hydrogels offer a highly efficient and sustainable solution, making them particularly well-suited for the remediation of antibiotic contamination.

### 3.1. Adsorption Performance Toward Tetracyclines

Owing to their complex amphoteric dissociation behavior and multiple active sites, the broad class of antibiotics known as tetracyclines (TCs) requires adsorbents with superior mechanical stability, broad pH adaptability, and high selectivity [47]. It is important to note that, while the literature often refers to “tetracycline” generically, experimental studies frequently use specific chemical forms, such as tetracycline hydrochloride (TC-HCl), rather than the free base. This distinction is critical, as the salt form exhibits different solubility and initial pH characteristics, which directly dictate its protonation state (cationic, zwitterionic, or anionic) in solution and subsequently alter its affinity for the hydrogel matrix. To address the challenges of tetracycline removal, current research has primarily focused on four strategic directions: reinforcing the material framework, broadening pH tolerance, enhancing specific recognition via molecular imprinting, and achieving ultra-high capacities through metal–organic frameworks or enzymatic coupling. However, evaluating these advancements requires a critical examination of the trade-offs between adsorption capacity, synthesis complexity, and practical stability.

To address the bottlenecks of chitosan’s low mechanical strength and recycling difficulties, the construction of double-network structures and magnetic composites has emerged as an effective solution. Li et al. [48] utilized a deep eutectic solvent to fabricate a chitosan, cellulose, and polyacrylamide (CCP) double-network hydrogel, characterized by an interpenetrating structure comprising rigid “chitosan-cellulose” and flexible “polyacrylamide” networks. Under acidic conditions, adsorption was proposed to be driven by electrostatic attraction between the protonated amino groups (NH_3_^+^) of chitosan and the dissociated phenolic hydroxyl groups (O^−^) of tetracycline. The hydrogel exhibited a compressive strength equivalent to 2.8 times that of a single network system. Furthermore, its high swelling ratio (955–1377%, dependent on temperature and cross-linking degree) provided abundant adsorption sites, maintaining adsorption capacity retention rate of 84.3–93.4% even after five regeneration cycles. Building on this, Dehghani Firoozabadi et al. [49] introduced Fe_3_O_4_ nanoparticles into an oxidized cellulose/chitosan hydrogel via imine-bond cross-linking, thereby synthesizing a magnetic hydrogel with Fenton-like catalytic activity. The tetracycline removal efficiency increased significantly with increasing magnetic nanoparticle content and chitosan concentration and with decreasing pH. In the presence of hydrogen peroxide, the Fe_3_O_4_ nanoparticles not only endowed the hydrogel with magnetic separability but also were hypothesized to promote the generation of hydroxyl radicals (·OH) through a Fenton-like reaction under acidic conditions, especially at pH < 4. This suggests a synergistic “adsorption–oxidation” mechanism, in which the hydrogel matrix facilitates tetracycline adsorption while Fe_3_O_4_-mediated H_2_O_2_ activation contributes to oxidative degradation.

**Figure 2:**
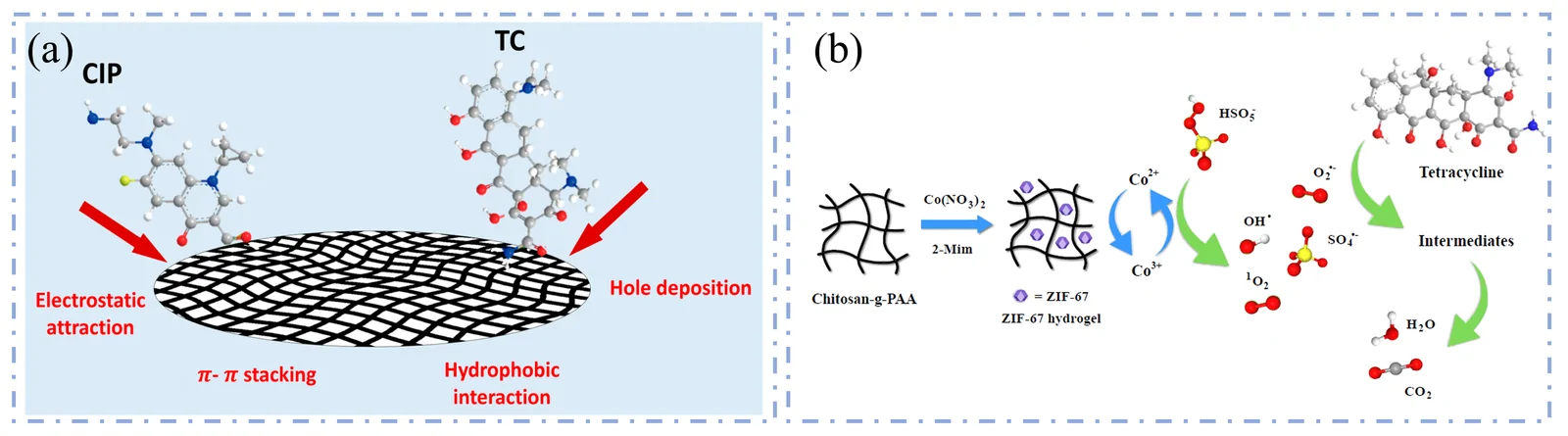
Targeted adsorption and catalytic degradation of tetracycline using engineered chitosan composites. (**a**) Schematic of the adsorption mechanism for SA/CMCS hydrogel microbeads. Reproduced with permission from Ref. [51]. Copyright 2025, MDPI. (**b**) Degradation of TC via PMS activation by ZIF-67 synthesized in situ within a chitosan-g-PAA hydrogel. Reproduced with permission from Ref. [55]. Copyright 2024, RSC Publishing.

To circumvent the limitation of chitosan deprotonation and subsequent inactivation in alkaline environments, chemical modification and functional integration offer novel pathways. Ranjbari et al. [50] incorporated an ionic liquid (trioctylmethylammonium chloride, TCMA) into the chitosan backbone, significantly broadening the pH range of applicability via an ion-exchange mechanism. Their study demonstrated that the material remained highly efficient across a pH range of 5–11. In evaluating the adsorption behavior, the process was found to follow pseudo-first-order kinetics—a mathematical model indicating that the adsorption rate is primarily dependent on the availability of unoccupied physical sites rather than chemical bonding, often suggesting a diffusion-controlled or physisorption-dominated process. Furthermore, the data fit the Langmuir isotherm model, which assumes that adsorption occurs as a single layer (monolayer) on a surface with a finite number of identical, homogeneous sites. This model is crucial for predicting the theoretical maximum adsorption capacity. The adsorption process followed pseudo-first-order kinetics and the Langmuir monolayer model; at an initial concentration of 50 mg/L and 45 °C, a removal efficiency of 90% was achieved within 45 min, with a maximum adsorption capacity of 22.42 mg/g. Although this adsorption capacity is relatively modest, the material’s stability over a wide pH window offers significant adaptability advantages for practical wastewater treatment. In a contrasting approach, Qian et al. [51] developed sodium alginate (SA) and carboxymethyl chitosan (CMCS) hydrogel microbeads fabricated via an electrostatic spray technique as targeted adsorbents for tetracycline (TC) and ciprofloxacin (CIP) antibiotics. Fourier-transform infrared spectroscopy confirmed that the adsorption process is predominantly driven by electrostatic interactions between the antibiotic molecules and the hydrogel network. Batch experiments revealed that maximum adsorption was achieved at pH 7 and 25 °C, with higher temperatures negatively impacting performance by weakening these electrostatic forces. The adsorption behavior of both antibiotics aligned with the pseudo-second-order kinetic model, while equilibrium data for single and binary systems were best described by the Freundlich and Temkin isotherms. In binary solutions, TC adsorption decreased due to strong competition with CIP for active sites. Notably, smaller microbeads (~400 µm) significantly outperformed larger variants (~2000 µm) by providing a greater surface area and more available interaction sites. Ultimately, these results highlight the strong potential of SA/CMCS hydrogel microbeads as highly effective, scalable biomaterials for remediating antibiotic-contaminated wastewater (Figure 2a).

To address the challenge of competitive adsorption from co-existing ions (e.g., Ca^2+^ and Mg^2+^) in real wastewater, molecularly imprinted polymers (MIPs) enhance selectivity by constructing specific recognition cavities. Kurczewska et al. [52] compared chitosan/halloysite MIP hydrogels tailored via different synthesis strategies. They found that microspheres optimized under diluted conditions exhibited thinner polymer layers and enhanced thermal stability, achieving a maximum adsorption capacity of 178.05 mg/g (slightly higher than 175.24 mg/g obtained by conventional methods). In a subsequent study [53], the matrix was replaced with alginate (ALG). The resulting ALG Hal-deposited polymer microspheres exhibited an increased adsorption capacity of 281 mg/g (a 58% improvement over the CS-based counterpart). This enhancement was hypothesized to result from the higher density of carboxyl groups in ALG, which facilitated the formation of a more robust hydrogen bond network with the hydroxyl and amino groups of TC. Both studies suggested that the imprinted cavities enabled specific recognition of TC via a mechanism combining “pore size matching” and “multi-site synergy” (involving π-π stacking, electrostatic attraction, and hydrogen bonding). However, it is crucial to acknowledge that in these and many similar studies, such specific interactions are frequently inferred from indirect spectroscopic evidence (e.g., post-adsorption shifts in FTIR or XPS spectra) rather than direct in situ observation. Furthermore, the incorporation of halloysite nanotubes not only provided physical support but also utilized the silanol groups within their lumens to anchor the adsorbate, effectively mitigating the secondary release of antibiotics into groundwater.

To pursue the ultimate breakthrough in adsorption capacity and the complete mineralization of pollutants, metal–organic framework (MOF) compositing and bio-enzymatic technologies have emerged as frontier directions. Guo et al. [54] constructed a Co/Zn bimetallic MOF (UiO-66)/biochar/chitosan composite aerogel. By leveraging a hierarchical pore structure (mesopores 2–5 nm, macropores > 100 nm) and metal coordination centers, the material achieved a TC adsorption capacity of 1693.45 mg/g—approximately an 80-fold increase over that of pure chitosan hydrogels. This high uptake was characterized as a monolayer chemisorption process. In adsorption literature, such a conclusion is typically drawn when experimental data best fit the pseudo-second-order kinetic model—which suggests that chemical interactions, such as electron sharing or exchange, dictate the rate-limiting step—alongside the Langmuir isotherm. This monolayer chemisorption process was deduced from indirect evidence to be primarily mediated by the coordination of Lewis acid sites (Co^2+^/Zn^2+^) to the ketone carbonyl groups of TC, along with π-π stacking between aromatic rings. Notably, the material retained nearly 80% of its efficiency after 10 regeneration cycles. Distinct from physical enrichment strategies, Nachaichot et al. [55] to enable the efficient catalytic degradation of tetracycline, a composite hydrogel was fabricated by synthesizing zeolitic imidazolate framework-67 (ZIF-67) in situ within a chitosan-grafted poly(acrylic acid) (chitosan-g-PAA) network. This structure effectively activated peroxymonosulfate (PMS) through Co^2+^/Co^3+^ redox cycling, generating SO_4_^−^, HO, O_2_^−^, and ^1^O_2_ as the primary reactive species for degradation. The composite hydrogel exhibited rapid catalytic performance, completely degrading TC within 30 min under optimal conditions (1 g/L catalyst and PMS) and demonstrating broad applicability across a wide pH range (5–9). Furthermore, it maintained excellent practical viability, retaining over 78% degradation efficiency in real water samples containing competing anions. Antibacterial tests against E. coli confirmed that the resulting TC degradation products were nontoxic. Additionally, the hydrogel demonstrated strong versatility, effectively degrading other antibiotics (ciprofloxacin and norfloxacin) and completely removing various organic dyes. Capable of simple recovery via filtration, the composite hydrogel maintained robust catalytic activity across five consecutive regeneration cycles (Figure 2b).

### 3.2. Adsorption Performance for Quinolone Antibiotics

Quinolone antibiotics (e.g., ciprofloxacin and norfloxacin (NOR)), characterized by their zwitterionic structures and multi-aromatic ring skeletons, impose differentiated requirements on the pore structure, functional group density, and catalytic activity of adsorbent materials [56]. Current research revolves around the following four directions: incorporating high-specific-surface-area components such as MOFs and metal oxides to break through adsorption capacity limits; utilizing biochar compositing to reduce costs and enhance environmental adaptability; enhancing pH responsiveness and continuous processing capabilities through functional group grafting or membrane technology; and constructing “adsorption–degradation” dual-function systems to achieve pollutant mineralization.

To overcome chitosan’s low specific surface area (<10 m^2^/g), the incorporation of inorganic components, such as metal–organic frameworks (MOFs), has emerged as a predominant strategy to enhance adsorption capacity. Yang et al. [57] fabricated a chitosan (CS) double-network hydrogel synergized with reduced graphene oxide (rGO) and ZIF-67 (rGO@ZIF-67@CS). This material exhibited a remarkable maximum adsorption capacity of 1890.32 mg/g for NOR at pH 5 and 1685.26 mg/g for tetracycline at pH 4. The underlying mechanisms were proposed to involve (i) the enrichment of NOR molecules via pore filling within the ZIF-67 micropores (1.16 nm); (ii) π–π stacking interactions inferred from the structural compatibility between the sp^2^ carbon domains of rGO and the quinolone rings; and (iii) hydrogen bonding between the amino groups of chitosan and the carboxyl groups of NOR, as suggested by FTIR spectral shifts. Notably, in a binary system, the material achieved simultaneous removal efficiencies of 92.68% for TC and 82.46% for NOR. While the high adsorption capacity is compelling, the evidence for the specific contribution of each mechanism remains indirect. The simultaneous presence of multiple components (rGO, ZIF-67, CS) makes it challenging to deconvolute their individual roles, and the proposed π–π and hydrogen bonding interactions are based on post-adsorption characterization rather than direct proof of binding during adsorption. Similarly, adopting the MOF strategy, Guesmi et al. [58] developed chitosan/alginate beads reinforced with magnetic selenium-based MOFs (MSe-MOF, specific surface area 420 m^2^/g), denoted as MSCA. This material achieved an adsorption capacity of 440 mg/g for CIP. Thermodynamic analysis indicated an entropy-driven endothermic process (ΔH° > 0, ΔS° > 0), with chemisorption playing the dominant role. Beyond MOFs, Li et al. [59] utilized a microwave-assisted method to load hexagonal sheet-like MgO onto a biochar/chitosan matrix (BC-MgO-CS). The material attained a CIP adsorption capacity of 1678.9 mg/g. The mechanism was primarily attributed to the complexation of surface Lewis base sites (O^2−^) on MgO with the carboxyl groups of CIP, as well as to electrostatic attraction between Mg^2+^ and the ketone groups of CIP. A critical consideration for such metal oxide-based systems is the potential for metal ion (e.g., Mg^2+^) leaching during adsorption or regeneration, which could pose a secondary contamination risk—a factor often underreported in high-adsorption capacity performance studies.

Compared with the high synthesis costs associated with MOFs, biochar-based composites offer significant economic and resource-sustainability advantages. Afzal et al. [60] fabricated a humic acid-coated biochar/chitosan hydrogel (HBCB), exhibiting a maximum CIP adsorption capacity of 154.89 mg/g. The adsorption mechanism was suggested to rely on π–π electron donor-acceptor interactions between the aromatic structure of biochar and CIP, hydrogen bonding, and hydrophobic interactions. However, in the presence of Na_3_PO_4_, the adsorption capacity decreased by 23.92%, suggesting that PO_4_^3−^ ions compete with CIP for protonated amino groups on chitosan, thereby weakening electrostatic attraction. This competitive experiment provides stronger, more direct evidence for the electrostatic component of the mechanism than spectroscopic inferences alone. Subsequently, the same research group [61] further validated the broad-spectrum applicability of HBCB, reporting maximum adsorption capacities for NOR, lomefloxacin (LOM), and enrofloxacin (ENR) of 38.08 mg/g, 25.03 mg/g, and 29.72 mg/g, respectively. Although the adsorption capacity was lower than that of MOF-based materials, HBCB maintained a net adsorption capacity above 47 mg/g after four cycles, demonstrating excellent cost-effectiveness.

To mitigate the electrostatic repulsion arising from the negative charge of quinolones under neutral to alkaline conditions, introducing anion-responsive functional groups has become a key optimization strategy. Wang et al. [62] synthesized an acrylic acid-grafted hydrogel, leveraging abundant COO^−^ groups to achieve a highly sensitive pH response. At pH 3, the material exhibited adsorption capacities of 267.7 mg/g for CIP and 387.7 mg/g for ENR, while retaining an adsorption rate of over 85% after five cycles. In continuous-flow treatment scenarios, membrane technology has emerged as a research hotspot due to its high flux and selectivity. Tang et al. [63] innovatively incorporated a chitosan/covalent organic framework (CH@COF) combined with tannic acid-Fe^3+^ as an interlayer within nano-filtration membranes. Compared to the pristine polyamide membrane (flux: 8.74 L/m^2^/h/bar), the composite membrane increased water flux to 16.17 L/m^2^/h/bar (an 85% enhancement), while achieving rejection rates of 94.89%, 99.07%, and 99.10% for NOR, CIP, and OFL, respectively. The high rejection performance was attributed to size sieving through the ordered pores of the COF, hydrogen bonding between the phenolic hydroxyl groups of tannic acid and quinolones, and coordination interactions between Fe^3+^ and carboxyl groups. Furthermore, the membrane demonstrated a flux recovery ratio of nearly 98% against protein and polysaccharide foulants, offering an efficient solution for continuous flow antibiotic wastewater treatment.

Traditional adsorption methods face the dilemma of requiring thermal desorption or solvent regeneration upon saturation, often leading to secondary pollution. To achieve in situ mineralization of pollutants, Afzal et al. [64] developed TiO_2_/biochar-loaded chitosan microspheres (TBCB), integrating adsorption with sonophotocatalytic degradation. TBCB achieved efficient CIP degradation (85.23%) under ultrasonic power, generating active species such as •O_2_^−^, h^+^, and •OH. Quenching experiments revealed that adding isopropanol (a •OH scavenger) resulted in the largest decrease in degradation rate, providing evidence for the critical role of hydroxyl radicals. This type of quenching experiment provides more direct mechanistic insight into degradation pathways than the indirect characterization often used for adsorption mechanisms. However, strong electrolytes inhibited catalytic activity, and the material maintained 62% efficiency after four cycles. This highlights a common limitation: the performance of integrated systems is often optimized under controlled, single-pollutant conditions, and their robustness in multi-component, variable ionic-strength environments remains a key challenge for practical application.

### 3.3. Removal of Sulfonamides and Other Antibiotics

Sulfonamide antibiotics (SAs), such as sulfamethoxazole (SMX) and sulfamethazine (SMZ), are characterized by stable sulfonamide groups (-SO_2_NH-) and weak acidity (pKa~5–7). Consequently, they exist primarily in anionic forms at neutral pH, imposing specific requirements on the charge density, pore-size compatibility, and redox activity of adsorbent materials. In response to these challenges, current research focuses on four main strategies: (1) incorporating photosensitive materials (e.g., g-C_3_N_4_, Embedding silver nanoparticles (AgNPs)) to enable in situ regeneration of adsorption sites and trace detection via surface-enhanced Raman scattering; (2) constructing differential recognition systems for hydrophilic and hydrophobic antibiotics through interpenetrating polymer networks and charge modulation; (3) optimizing pore structures via the intercalation effect of layered double hydroxides (LDH) to significantly enhance adsorption capacity; and (4) coupling with Advanced Oxidation Processes (AOPs) to achieve complete mineralization of pollutants and the inactivation of antibiotic resistance genes (ARGs).

The introduction of photosensitive materials enables low-cost regeneration of adsorbents and endows them with detection capabilities. Zhou et al. [65] prepared a g-C_3_N_4_-embedded CS/PVA composite hydrogel that adsorbed SMX at pH 5. The interaction was primarily proposed to be driven by π-π stacking between the triazine rings of g-C_3_N_4_ and the benzene rings of SMX, an inference based on the structural compatibility of the aromatic systems. Using the photocatalytic ability of g-C_3_N_4_, adsorption sites could be reactivated upon UV exposure. In contrast, the CS/CN/Ag flexible substrate developed by Luo et al. [66] integrated broader functionalities. Through in situ reduction, spherical AgNPs (diameter 6–12 nm, spacing < 20 nm) were loaded onto a CS/carbon nitride aerogel. While adsorbing SMX, the material utilized the surface plasmon resonance effect of Ag to achieve surface-enhanced Raman scattering (SERS) detection. The adsorption rate for SMX reached 83.06% within 20 min, with a SERS limit of detection (LOD) as low as 7.46 × 10^−9^ mol/L (enhancement factor of 3.3/times 10^2^). Also, after 20 min of irradiation, the SMX degradation rate reached 99.22%. This enhanced efficiency was attributed to hot electrons from Ag surface plasmon excitation being injected into g-C_3_N_4_, a mechanism supported by the observed performance enhancement but not directly proven at the molecular level. The material maintained excellent performance over six cycles, realizing a “closed loop” from trace detection to efficient remediation; however, the potential biological toxicity of Ag requires careful consideration in practical applications.

Given the complex coexistence of multiple antibiotics and endocrine-disrupting chemicals in actual effluents, the development of broad-spectrum adsorbents is paramount (Table 1). Zhou et al. [67] engineered a powder activated carbon-modified CS/PVA hydrogel capable of dual-mode adsorption: hydrophilic sulfamethoxazole was primarily sequestered via hydrogen bonding with the hydroxyl/amino groups of CS (maximum adsorption capacity of 9.1 mg/g at pH 4), whereas hydrophobic bisphenol A (BPA) preferentially partitioned into the hydrophobic pores of PAC (maximum adsorption capacity of 64.6 mg/g, stable across pH 2–9). The adsorption isotherms fit the Langmuir model well, and the material maintained its high capacities (SMX > 7 mg/g, BPA > 50 mg/g) even in real water matrices containing humic acid and interfering ions. To further enhance functionality, temperature-responsive systems were explored. A poly(N-isopropylacrylamide) (PNIPAM)/CS interpenetrating network (IPN) hydrogel demonstrated thermally tunable adsorption [68]: increasing the temperature from 25 °C to 35 °C tripled the uptake of hydrophobic BPA, while sulfamethazine adsorption remained virtually unchanged. Equilibrium was achieved within 5 min for both solutes, driven by distinct mechanisms: SMZ relied on electrostatic attraction (between anionic -SO_2_NH^−^ and cationic -NH_3_^+^), while BPA uptake was governed by hydrophobic inclusion into the cavities formed by collapsed PNIPAM chains. However, deconvoluting the exact contribution of hydrophobic effects versus potential changes in hydrogen bonding or pore structure with temperature remains challenging based on the presented data. Regarding the precise modulation of charge density, Aranda et al. [69] synthesized cationic hydrogel particles, for where the optimal adsorption pH for amoxicillin and trimethoprim was determined to be 6.0 and 4.0, respectively. This disparity stems from speciation: at pH 6, amoxicillin (AMX) exists as a zwitterion (-COO^−^/-NH_3_^+^), maximizing electrostatic attraction between its anionic carboxylate moiety and the quaternary ammonium groups of the CHPs; in contrast, TMP requires protonation (-NH_2_^+^) at pH 4 for effective interaction. The material removed more than 90% of AMX at room temperature, and thermodynamic analysis showed that the process was spontaneous and endothermic (ΔH° > 0). Increased ionic strength—particularly the presence of mono- and divalent ions—significantly impeded the interaction, thereby confirming that electrostatic attraction plays a dominant role in the adsorption mechanism.

The intercalation effects of Layered Double Hydroxides and the dispersion of nanoparticles are pivotal strategies for optimizing the pore architecture of hydrogel matrices. Samani et al. [70] incorporated EDTA-intercalated LDH into an O-carboxymethyl chitosan (O-CMCS)/pectin hydrogel. The tridentate coordination of EDTA facilitated the uniform peeling of LDH lamellae, resulting in a more uniform pore-size distribution. This structural modification enhanced thermal stability by 2.5-fold and increased oxacillin (BP) adsorption capacity by 3–4 times compared with conventional hydrogels. The uptake mechanism was described as being governed by coupled Fickian diffusion and polymer chain relaxation. Mosaffa et al. [71] developed a Ni-Al LDH-modified biochar/chitosan double-crosslinked hydrogel that could retain substantial amounts of material. They found that it could bind a large amount of erythromycin (763 mg/g) and amoxicillin (835 mg/g). The mechanism was proposed to rely on a synergistic interplay: coordination between Lewis acid sites (Ni^2+^) on the LDH and antibiotic moieties, coupled with π-π stacking between the biochar and ERY’s conjugated structure. While the high adsorption capacity is clear, the evidence for this specific synergy is indirect, based on the presence of these components and characterization data. The potential for Ni^2+^ leaching—a critical secondary pollution risk—is an important but often underreported consideration for LDH-based adsorbents. At a concentration of 20 mg/L, removal efficiencies reached 94.6% and 98.2%, respectively, with a regeneration efficiency of ≥88% retained after seven cycles (Table 1). Alternatively, to address synthesis efficiency and end-of-life safety, Mahmoud et al. [72] used microwave cross-linking to fabricate a nanosilica/peel biochar/chitosan composite (BPNB-NSiO_2_-Chit Hgel). The material efficiently adsorbed ERY in a single layer because its nanostructure was uniform (particle size: 22.48–26.23 nm). A distinct advantage of this composite stems from the nano-SiO_2_, which provides additional silanol moieties (-Si-OH) as hydrogen-bonding sites. Furthermore, thermogravimetric analysis confirmed that the spent adsorbent decomposes into stable SiO_2_, ensuring environmental safety upon disposal.

To achieve complete mineralization and avoid secondary pollution, He et al. [73] synthesized a core–shell Fe@nitrogen-doped carbon nanocomposite templated by carboxymethyl chitosan for activating peroxymonosulfate. This system achieved rapid, complete SMX degradation across a broad pH range and under high-salinity conditions. Mechanistic investigations identified singlet oxygen (^1^O_2_) as the proposed dominant reactive species utilizing a non-radical pathway, based on quenching experiments and electron paramagnetic resonance spectroscopy. Quenching experiments provide stronger direct evidence for specific degradation pathways than the indirect characterization often used for adsorption mechanisms. Beyond chemical degradation, the system inactivated antibiotic-resistant bacteria and intracellular ARGs. This establishes a promising holistic strategy; however, its long-term stability, the potential formation of toxic byproducts, and performance in real wastewater containing natural organic matter require further validation to assess practical feasibility.

**Table 1:** Comparing the Adsorption and Degradation Performance of Antibiotics by Chitosan-Based Composite Hydrogels.

Adsorbent Material/System	Target Pollutant	Max. Adsorption Capacity (mg/g)	Operating Conditions (pH, Time, Temp)	Regeneration Performance	Dominant Mechanism(s)	Ref.
CCP double-network hydrogel (DES-based)	TC	/	Acidic conditions	5 cycles (84.3–93.4% retention)	Electrostatic attraction (NH_3_^+^ and O^−^)	[48]
Oxidized cellulose/CS/Fe_3_O_4_ magnetic hydrogel	TC	/	pH < 4, H_2_O_2_ presence	Magnetic separation	Synergistic adsorption-oxidation (Fenton-like, ·OH)	[49]
TCMA-modified chitosan (Ionic liquid)	TC	22.42	pH 5–11, 45 min, 45 °C	High stability over a wide pH window	Ion-exchange, pseudo-first-order, Langmuir monolayer	[50]
CS/halloysite MIP hydrogels	TC	178.05	Diluted synthesis conditions	/	Specific recognition, pore matching, multi-site synergy	[52]
Co/Zn MOF (UiO-66)/biochar/CS aerogel	TC	1693.45	/	10 cycles (~80% retention)	Monolayer chemisorption, Lewis acid coordination, π-π stacking	[54]
rGO@ZIF-67@CS double-network hydrogel	NOR/TC	1890.32 (NOR)/1685.26 (TC)	pH 5 (NOR), pH 4 (TC)	/	Pore filling, π-π stacking, H-bonding	[57]
CS/alginate beads + MSe-MOF	CIP	440	/	/	Entropy-driven endothermic chemisorption	[58]
BC-MgO-CS composite	CIP	1678.9	/	/	Lewis base/acid complexation, electrostatic attraction	[59]
Humic acid-coated biochar/CS (HBCB)	CIP	154.89	/	4 cycles (>47 mg/g net adsorption capacity)	π-π EDA interactions, H-bonding, hydrophobic, electrostatic	[60]
Acrylic acid-grafted hydrogel	CIP/ENR	267.7 (CIP)/387.7 (ENR)	pH 3	5 cycles (>85% retention)	Electrostatic attraction (anion-responsive COO^−^)	[62]
CH@COF + TA-Fe^3+^ interlayer membrane	NOR, CIP, OFL	>94% rejection	Continuous flow	98% flux recovery	Size sieving, H-bonding, Fe^3+^ coordination	[63]
TiO_2_/biochar-loaded CS microspheres	CIP	85.23% degradation	Ultrasonic power	4 cycles (62% efficiency)	Sono-photocatalytic degradation (•O_2_^−^, h^+^, •OH)	[64]
CS/carbon nitride aerogel + AgNPs	SMX	83.06% (ads)/99.22% (deg)	20 min contact/UV	6 cycles	SERS detection, hot electron injection, π-π stacking	[66]
PAC-modified CS/PVA hydrogel	SMX/BPA	9.1 (SMX)/64.6 (BPA)	pH 4 (SMX), pH 2–9 (BPA)	Stable in real water matrices	H-bonding (SMX), hydrophobic partition (BPA)	[67]
PNIPAM/CS IPN hydrogel	SMZ/BPA	/	5 min, 25 °C to 35 °C	/	Electrostatic (SMZ), hydrophobic inclusion (BPA)	[68]
Cationic hydrogel particles (CHPs)	AMX/TMP	>90% removal	pH 6.0 (AMX), pH 4.0 (TMP)	/	Electrostatic attraction (pH-dependent speciation)	[69]
Ni-Al LDH-modified biochar/CS hydrogel	ERY/AMX	763 (ERY)/835 (AMX)	20 mg/L initial conc.	7 cycles (≥88% retention)	Lewis acid coordination (Ni^2+^), π-π stacking	[71]
Fe@N-doped carbon/CMCS nanocomposite	SMX	Complete degradation	Broad pH, high salinity	/	PMS activation, singlet oxygen (^1^O_2_) non-radical pathway	[73]

Note: TC, Tetracycline; CIP, Ciprofloxacin; NOR, Norfloxacin; ENR, Enrofloxacin; OFL, Ofloxacin; SMX, Sulfamethoxazole; SMZ, Sulfamethazine; AMX, Amoxicillin; TMP, Trimethoprim; ERY, Erythromycin; CS, Chitosan; MIP, Molecularly imprinted polymer; MOF, Metal–organic framework; LDH, Layered double hydroxide; PAC, Powder activated carbon; /, Data not explicitly detailed in the text. Adsorption mechanisms derived from mathematical modeling (e.g., kinetics, isotherms) are identified as proposed or inferred. In contrast, molecular interactions are considered experimentally verified only when supported by direct spectroscopic evidence (e.g., FTIR, XPS).

## 4. Application of Chitosan-Based Hydrogels for Dye Removal

The substantial heterogeneity in the charge characteristics and molecular structures of dyes in textile effluents necessitates meticulous regulation of the functional group chemistry and pore topology of adsorbents. Chitosan-based hydrogels employ targeted modification strategies to achieve high-efficiency removal across variable dye classes:

(1) For cationic dyes: The inherent electrostatic repulsion between the polycationic chitosan backbone and positively charged dyes is mitigated by grafting anionic moieties (e.g., carboxyl or sulfonic groups) or compositing with anionic materials, effectively reversing the surface charge to favor adsorption.
(2) For anionic dyes: The mechanism leverages the intrinsic electrostatic attraction provided by the protonated amino groups (-NH_3_^+^) of chitosan, while cross-linking strategies are simultaneously employed to reinforce the hydrogel’s structural integrity and prevent dissolution.
(3) For reactive dyes and complex mixed systems: Multimodal interaction mechanisms—integrating hydrogen bonding, π-π stacking, and hydrophobic interactions—are engineered to achieve selective adsorption and separation in competitive environments.

### 4.1. Removal of Cationic Dyes

The removal of cationic dyes (e.g., Crystal Violet (CV), Methylene Blue (MB)) by pristine chitosan is often restricted (capacities typically <50 mg/g) due to the strong electrostatic repulsion between the positively charged dye molecules and the protonated amino groups (-NH_3_^+^) of the chitosan backbone. To resolve this limitation, current modification strategies primarily include: Anionic functionalization: Introducing anionic groups (-COO^−^, -PO_3_^2−^) or surfactants to neutralize repulsive forces and enhance electrostatic attraction; Magnetic compositing: Incorporating magnetic particles (e.g., Fe_3_O_4_) to facilitate rapid solid–liquid separation while significantly boosting adsorption capacity to levels exceeding 500 mg/g; Nanoparticle loading: Embedding silver nanoparticles to endow the adsorbent with dual capabilities of dye removal and antibacterial activity.

**Figure 3:**
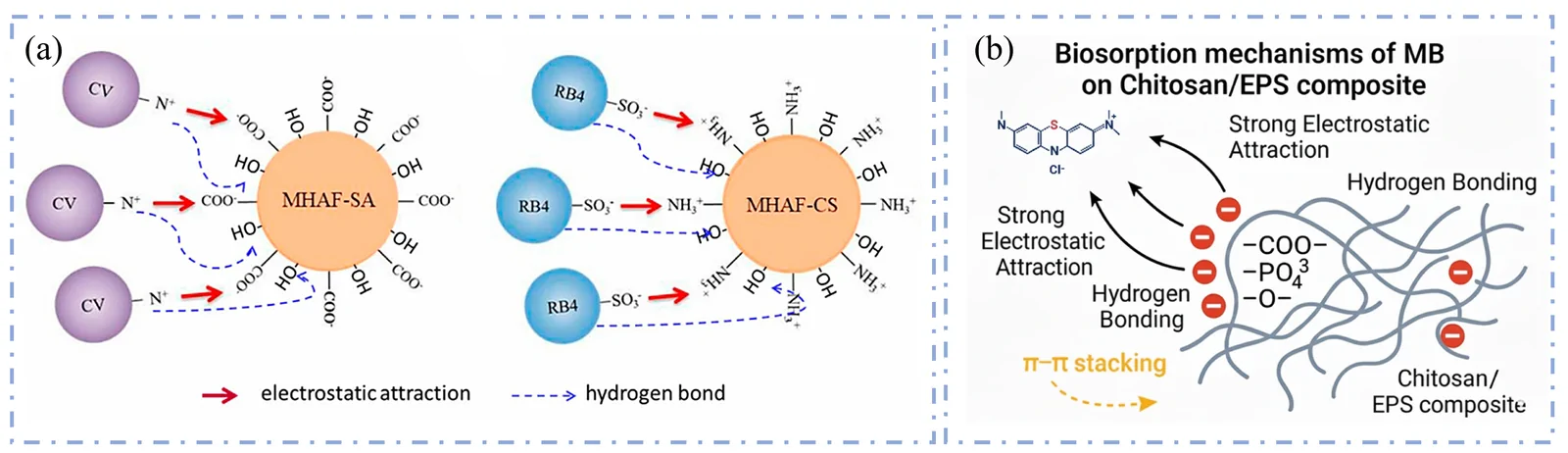
Design of cross-linked hydrogels and their interaction mechanisms with cationic dyes. (**a**) Adsorption mechanism of CV onto MHAF-SA and RB4 onto MHAF-CS. Reproduced with permission from Ref. [76]. Copyright 2021, MDPI. (**b**) Schematic representation of methylene blue (MB^+^) biosorption by a chitosan/EPS composite. Reproduced with permission from Ref. [79]. Copyright 2026, Springer Nature.

The functionalization of chitosan with anionic groups effectively reverses the surface charge, facilitating the high-efficiency capture of cationic dyes. Nakhjiri et al. [74] prepared a maleated chitosan hydrogel grafted with acrylic acid and phosphonic acid. The adsorption process was found to be spontaneous and endothermic, fitting the Redlich-Peterson isotherm model. This model incorporates features of both Langmuir and Freundlich isotherms, suggesting a versatile, mixed adsorption mechanism across varying concentrations rather than ideal monolayer coverage. The material achieved maximum adsorption capacities of 64.56 mg/g for Crystal Violet and 66.89 mg/g for Methylene Blue, with stable performance over four cycles following ethanol desorption. Similarly, the incorporation of anionic surfactants enhances adsorption through dual electrostatic and hydrophobic interactions. Pal et al. [75] developed chitosan gel beads embedded with sodium dodecyl sulfate (SDS). A notable synergistic effect was noted: the anionic head groups (-OSO_3_^−^) of SDS attracted cationic CV^+^ through electrostatic forces, while the hydrophobic alkyl chains (C_12_H_25_-) interacted with the benzene rings of CV through hydrophobic forces. The system reached a maximum adsorption capacity of 76.9 mg/g, determined by pseudo-second-order kinetics. This kinetic behavior indicates that chemisorption—involving valence forces through the sharing or exchange of electrons between the adsorbent and the dye—acts as the rate-limiting step, rather than simple mass transfer. After saturation, extended desorption facilitated the selective release of approximately 47% of the CV while preserving the SDS, thus permitting the functional regeneration of the adsorbent. Comparative studies on polysaccharide backbones demonstrate that the inherent anionic characteristic of sodium alginate (SA) confers a markedly greater affinity for cationic dyes than CS. Ma et al. [76] effectively removed organic dyes from aqueous solutions; novel hydrogel beads based on the husk of agarwood fruit (HAF) were synthesized by incorporating either sodium alginate (MHAF-SA) or chitosan (MHAF-CS). FTIR analysis confirmed the successful integration of their functional groups. Batch experiments, evaluating initial pH, adsorbent dosage, and contact time, demonstrated that MHAF-SA and MHAF-CS were highly effective for the targeted removal of crystal violet (CV) and reactive blue 4 (RB4), respectively. Kinetic studies revealed that CV adsorption onto MHAF-SA followed a pseudo-second-order model, whereas RB4 adsorption onto MHAF-CS fit a pseudo-first-order model. Both adsorption processes aligned with the Langmuir isotherm, exhibiting impressive maximum adsorption capacities of 232.56–370.37 mg/g for CV and 156.25–270.27 mg/g for RB4. Furthermore, both hydrogel beads displayed excellent adsorption selectivity and maintained high removal efficiencies even after five adsorption–desorption cycles, proving their strong potential and reusability for practical wastewater treatment (Figure 3a).

The incorporation of Fe_3_O_4_ magnetic particles simultaneously enhances adsorption capacity and enables magnetic separation, effectively resolving the recovery dilemma associated with traditional hydrogels in complex aqueous environments. Çavuşoğlu et al. [77] compared magnetic activated carbon (AC-Fe_3_O_4_) with its chitosan-coated counterpart (Chitosan-AC-Fe_3_O_4_). Upon coating with CS, the maximum adsorption capacity at 298 K surged from 266.57 mg/g to 505.87 mg/g, fitting a non-linear Freundlich model. Unlike models assuming a uniform single layer, the Freundlich fit characterizes multilayer adsorption on highly heterogeneous surfaces, where binding sites have varying affinities for the adsorbate. However, regeneration experiments revealed stability issues regarding the CS layer: over three cycles (using 1 M acetic acid for desorption), the desorption efficiency dropped from 75.35% to 41.87% for AC-Fe_3_O_4_ and from 64.63% to 27.84% for the composite. This decline suggests partial dissolution of the CS layer in the acidic medium or irreversible binding with the dye, necessitating improvements through cross-linking reinforcement or desorbent optimization. To balance high adsorption capacity with multi-functionality, the integration of mixed metal oxides has emerged as a new trend. Sadat et al. [78] developed a gelatin-chitosan magnetic hydrogel integrated with ZnCr_2_O_4_/Fe_3_O_4_. The material had a specific surface area of 68.83 m^2^/g and achieved maximum capacities of 235.5 mg/g for MB and 195.5 mg/g for CV (removal rates > 97%). The material demonstrated distinct antibacterial rates against S. aureus (99.64%) and E. coli (98.25%), attributed to the slow release of Zn^2+^ and Cr^3+^. The present study highlights the potential for synergistic “adsorption–antibacterial” purification in dyeing wastewater treatment, which often contains high bacterial loads (10^5^–10^7^ CFU/mL). While integrating metal oxides provides valuable multi-functionality, the pursuit of entirely green, biodegradable materials remains a parallel priority to ensure environmental sustainability. Abd-Alla et al. [79] developed a novel, fully biodegradable composite of chitosan and Bacillus subtilis exopolysaccharides (EPS) for the biosorption of methylene blue. Fourier-transform infrared spectroscopy confirmed that dye removal was driven by electrostatic, hydrogen-bonding, and π–π interactions with specific functional groups (such as -OH, -NH, and -COO−) on the biosorbent. Batch experiments demonstrated that the composite achieved optimal removal at pH 6, reaching equilibrium rapidly within 30 min and yielding a decolorization rate of 71.6%, successfully outperforming pristine chitosan. The adsorption process was best described by the pseudo-second-order kinetic model and the Langmuir isotherm, indicating efficient monolayer adsorption with a maximum biosorption adsorption capacity of 14.26 mg/g (and calculated kinetic capacities reaching 45.87 mg/g). By leveraging the synergistic effects of renewable microbial EPS and chitosan without the use of harsh chemicals, this fast-acting, cost-effective composite shows strong potential for scale-up and integration into continuous-flow wastewater treatment systems (Figure 3b).

**Figure 4:**
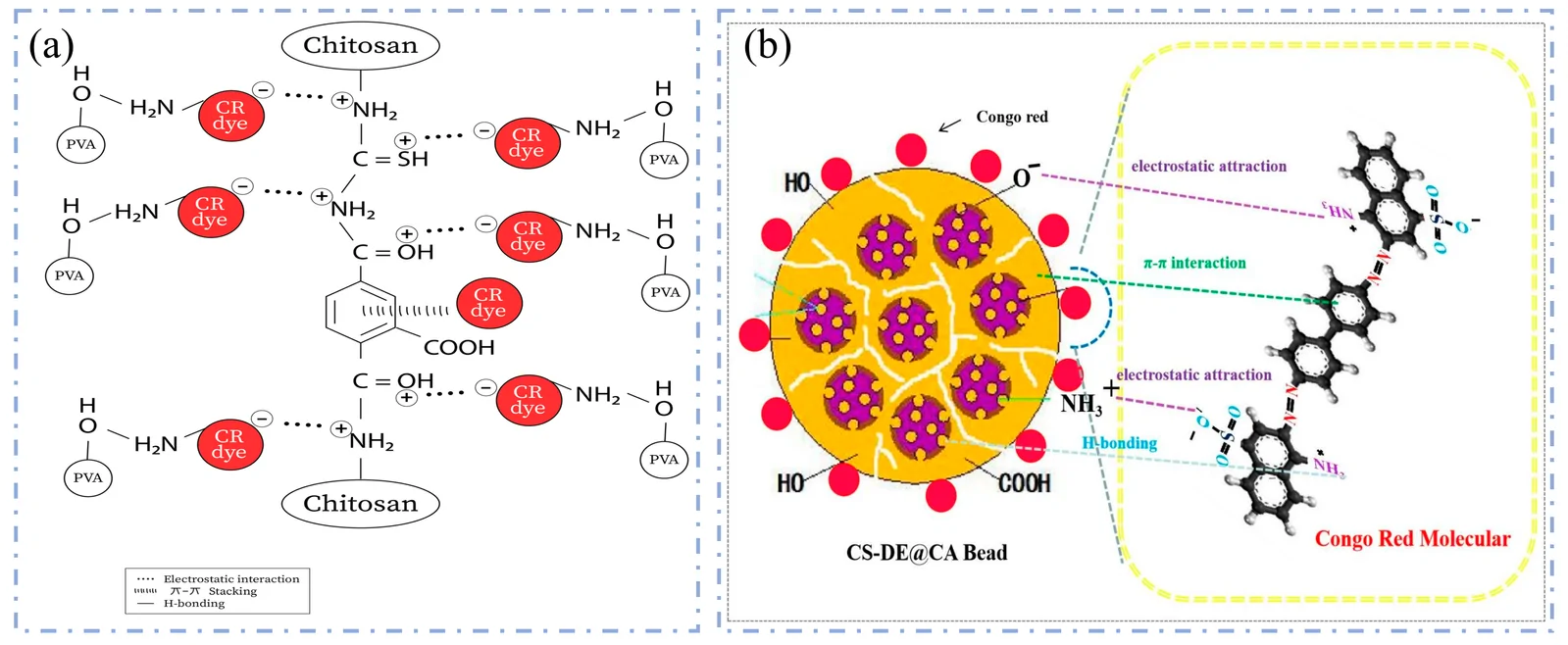
Remediation strategies for anionic dyes and mixed dye systems via adsorption and photocatalytic degradation. (**a**) Proposed adsorption mechanism for Congo red (CR) dye by the prepared hydrogels at pH 4. Reproduced with permission from Ref. [81]. Copyright 2023, MDPI. (**b**) Proposed mechanism for CR removal by CS-DE@CA composite beads. Reproduced with permission from Ref. [86]. Copyright 2023, MDPI.

The introduction of silver nanoparticles endows materials with antimicrobial properties and enhances dye adsorption through surface polarization effects. Hossein et al. [80] employed Reversible Addition-Fragmentation Chain Transfer polymerization to synthesize CS-based nanocomposite hydrogels in the presence of introduction of silver nanoparticles endows. The resulting material demonstrated spontaneous, endothermic adsorption and excellent antibacterial activity against Gram-negative bacteria. The blend ratio of CS with polyvinyl alcohol and the introduction of silver nanoparticles content differentially impact the removal efficiency for different dyes. Alfuraydi et al. [81] investigated the synergistic mechanisms between the CS/PVA ratio and AgNP loading. They discovered that the elimination of Congo Red (CR) increased with elevated CS and Ag concentrations (attaining 99.91% for the H31/Ag5% group), whereas the removal of Crystal Violet increased with higher PVA and Ag levels (reaching 94.7% for the same group). The adsorption data fitted the Freundlich model, reaffirming a mechanism of multilayer adsorption driven by interactions across heterogeneous surface sites. AgNPs contributed by providing additional binding sites and surface area, highlighting the material’s dual potential in both environmental remediation and biomedical applications (Figure 4a).

### 4.2. Removal of Anionic Dyes

The adsorption of anionic dyes (e.g., Congo Red and Acid Orange) by pristine chitosan is often limited, with capacities ranging from 30 to 50 mg/g, due to insufficient electrostatic interactions with the amino groups (-NH_3_^+^). Current modification strategies include: Quaternization (quaternary ammonium salt grafting) or magnetic compounding to enhance positive charge density, which can skyrocket the adsorption capacity to over 800 mg/g; MOFs or double-network designs can be incorporated to enhance structural stability and self-healing capabilities. The process can also be coupled with photocatalysis to achieve dye mineralization (degradation), which in turn eliminates secondary pollution.

The introduction of high-density quaternary ammonium cationic groups (-N^+^(CH_3_)_3_) can significantly reverse the surface charge of chitosan, thereby enabling ultra-high-capacity adsorption of anionic dyes. Liu et al. [82] prepared a semi-interpenetrating network (semi-IPN) hydrogel composed of chitosan and poly(dimethyldiallylammonium chloride-co-acrylamide) (CPDA). This material remained stable within a pH range of 4–10. Through the synergistic effect of electrostatic interactions and dye self-assembly, the hydrogel achieved an astonishing maximum adsorption capacity for Congo Red of 1803.507 mg/g (fitting the Hill model). The very high adsorption capacity was due to multilayer self-assembly of dye molecules on the quaternary ammonium sites, rather than simple monolayer adsorption. The material retained excellent performance even after six reuse cycles. Magnetic functionalization has been a breakthrough in efforts to recover traditional hydrogels from complex water environments. Patel et al. [83] employed in situ ultrasonic polymerization in conjunction with Fe_3_O_4_ integration to produce a pH-responsive magnetic chitosan-grafted copolymer hydrogel. This material possesses both high swelling adsorption capacity and magnetic separability. The maximum adsorption capacities for Methylene Blue and CR reached 1111.11 mg/g and 862.06 mg/g, respectively. The use of an external magnetic field enables efficient removal and separation of the dye-loaded hydrogel from the solution. The adsorption process followed the Freundlich isotherm and the pseudo-second-order kinetic model, demonstrating the synergistic advantages of active-group enrichment and magnetic recovery.

The high specific surface area of metal–organic frameworks and their coordination crosslinking with chitosan can synergistically enhance both adsorption capacity and structural stability. Jing et al. [84] fabricated a chitosan@UiO-67 metal–organic framework (CS@UiO-67) hydrogel, where covalent coordination between the hydroxyl groups of chitosan and the zirconium (Zr) ions in UiO-67 greatly improved the composite’s stability. The adsorption behavior was best described by the Langmuir isotherm and the pseudo-second-order kinetic model, indicating that dye uptake occurs as a uniform monolayer and that chemisorption is the rate-limiting step. Under these conditions, the composite achieved a remarkable maximum adsorption capacity of 1001.2 mg/g for Congo Red. Consistent with this chemisorption profile, the underlying adsorption mechanisms involved strong electrostatic interactions between the amino groups of CS and the sulfonate groups (-SO_3_^−^) of CR, as well as π-π stacking within the UiO-67 pores and hydrogen bonding networks. However, after four regeneration cycles, the removal efficiency decreased from 98% to 65%, primarily due to the irreversible occupation of these strong adsorption sites. Alternatively, Double-Network (DN) designs can enable materials to heal themselves by simultaneously employing two distinct crosslinking mechanisms. Zhang et al. [85] prepared a DN self-healing hydrogel by crosslinking carboxyethyl chitosan with oxidized sodium alginate and adding Ca^2+^ to form “calcium bridges.” This dual crosslinking strategy (“covalent bonds + ionic bonds”) increased the compressive strength to 1.2 MPa (compared to only 0.3 MPa for pure CS) and enabled the material to self-heal within 12 h after physical damage. This recovery is attributed to the dynamic coordination rearrangement of Ca^2+^. The monolayer adsorption capacities for MB and CR were 254.41 mg/g and 185.43 mg/g, respectively, offering promising potential for long-term applications.

Diatomite’s natural porous structure and the Lewis acid sites of rare-earth oxides provide low-cost adsorption solutions. Zhao et al. [86] developed diatomite composite gel beads (CS-DE@CA), which, despite their low cost, achieved a high removal efficiency of 89.9% (compared to 83.6% for the control). This improvement was attributed to the mesoporous structure of diatomite (pore size 2–50 nm), which provides additional mass-transfer pathways for dye molecules (Figure 4b). The maximum adsorption capacity was 48.42 mg/g (calculated using the Langmuir model). The adsorbent maintained satisfactory regeneration performance after four adsorption–desorption cycles. Conversely, doping with rare earth oxides can significantly accelerate adsorption kinetics. Bingöl et al. [87] prepared a Gd_2_O_3_-doped Ch-PVA-Gd hydrogel that exhibited ultrafast kinetics. At pH 6, with a 0.04 g dosage and 3% Gd_2_O_3_ doping, the material achieved over 99% removal of CR within 10 min, with a maximum adsorption capacity of 312.5 mg/g. This rapid kinetic performance is attributed to the Lewis acid sites of Gd^3+^, which accelerate dye coordination. While the material retains high efficiency even in high-concentration dye environments, its higher cost limits large-scale application. The introduction of graphene and its derivatives can synergistically enhance adsorption performance through π-π interactions and porous structures. Omidi et al. [88] synthesized a graphene/chitosan aerogel designed to remove both cationic and anionic dyes, demonstrating particularly high adsorption capacity for the anionic dye Congo Red. It achieved an adsorption capacity of 384.62 mg/g, with removal rates remaining near 100% over three cycles. To compare the differences between graphene oxide and graphene, Das et al. [89] prepared a GO/chitosan-PVA gel featuring good thermal stability and a porous structure. At pH 2, this gel removed 88.17% of CR, but at neutral pH, it removed only 81%. This performance was lower than that reported by Omidi, primarily due to experimental variations: Das used a significantly higher initial dye concentration (200 mg/L) compared to Omidi (50 mg/L). Furthermore, although the oxygen-containing functional groups (-COOH, -OH) of GO enhance hydrophilicity and thermal stability, the deprotonation of excessive -COOH groups at neutral pH creates negative charges. The result leads to electrostatic repulsion against anionic dyes, thereby weakening adsorption. Additionally, the study successfully predicted the hydrogel’s adsorption behavior using an Artificial Neural Network model.

While traditional adsorption methods can efficiently separate dyes, they essentially represent a process of “pollutant transfer,” necessitating subsequent desorption and regeneration treatments. The introduction of photocatalytic functionality enables the in situ mineralization of dyes following adsorption-enrichment, thereby achieving complete elimination. Jiang et al. [90] developed a Colloidal CdS sensitized nano-ZnO/chitosan (CdS@n-ZnO/CS) hydrogel that exhibited ultrafast degradation capabilities under sunlight irradiation. It achieved 95% removal of 5.0 mg/L CR in just 1 min and 94.34% removal of a high-concentration solution (100 mg/L) within 30 min. This exceptional degradation speed is attributed to the synergistic effect between the adsorption capacity of chitosan and the photocatalytic activity of ZnO and CdS. Radical scavenging experiments demonstrated that holes (h^+^) and superoxide radicals (·O_2_^−^) were the principal active species facilitating the degradation process. This “adsorption-photocatalysis” dual-function design realizes a qualitative leap from mere pollutant transfer to mineralization and degradation.

### 4.3. Removal of Metal Complex Dyes, Vat Dyes, and Direct Dyes

Compared with cationic and anionic dyes, metal complex dyes (e.g., cobalt phthalocyanine), vat dyes (e.g., indigo), and direct dyes (e.g., Direct Red 80) pose greater challenges to CS-based hydrogels due to their large molecular weights (600–1500 g/mol), complex conjugated structures, and frequent association with high-salinity wastewater. Current modification strategies include the use of aromatic crosslinkers to enhance π-π interactions and facilitate magnetic separation, as well as the use of Interpenetrating Polymer Network designs or biomass doping to improve swelling properties and adsorption capacity. Utilizing nano-filtration membranes to achieve effective dye/salt separation; Introducing synergy with MOFs or photocatalysis to realize mineralization and degradation.

Pristine chitosan typically exhibits limited affinity for metal complex dyes (e.g., cobalt phthalocyanine) and vat dyes (e.g., indigo), primarily due to steric hindrance and the lack of specific binding sites for their extended conjugated π-systems (containing 4–6 benzene rings). However, the incorporation of aromatic crosslinkers has been shown to enhance selective adsorption via π-π stacking interactions. Karimi et al. [91] pioneered a strategy utilizing 3,3′,4,4′-benzophenonetetracarboxylic dianhydride (BTCBDA) to crosslink CS, followed by ammonium sulfate modification, specifically for the sequestration of cobalt tetrasulfonated phthalocyanine (CoTsPc). The molecular recognition mechanism of this hydrogel includes (i) π-π stacking, characterized by face-to-face interactions between the benzophenone core of BTCBDA (three benzene rings) and the phthalocyanine ring of CoTsPc (four benzene rings), with a binding energy of approximately 15–25 kJ/mol; (ii) electrostatic attraction, mediated by the ion-exchange coordination of introduced sulfate anions (-SO_4_^2−^) with the Co^2+^ metal center of the dye; and (iii) pH-responsiveness. Under alkaline conditions (pH 8), the deprotonation of amino groups (-NH_2_) mitigates electrostatic repulsion towards sulfonate groups (-SO_3_^−^), yielding a removal efficiency of 98%. While the hydrogel maintains stable adsorption (>85% removal) over pH 4–8, adsorption capacity declines significantly at pH < 4, attributed to an altered electrostatic landscape arising from CS protonation (-NH_3_^+^). In distinct contrast, the removal of Indigo (IC, MW 262 g/mol), a non-ionic vat dye, exhibits optimal performance under acidic conditions (pH 4–5). Addressing this, Azadikhah et al. [92] synthesized a superparamagnetic composite hydrogel by crosslinking CS with benzophenonetetracarboxylic dianhydride (BTDA) and functionalized magnetic nanoparticles. The adsorption mechanism for IC is predominantly governed by (i) hydrophobic interactions, where the non-polar indigo moiety binds to the hydrophobic microdomains of the BTDA aromatic rings, and (ii) hydrogen bonding between the N–H groups of IC and the hydroxyl groups of CS (bond energy ~10–20 kJ/mol). Specifically, at pH 4, IC remains in a neutral molecular state, thereby maximizing hydrophobic interactions and achieving a 98.9% removal rate. The adsorption process follows the Langmuir isotherm, indicating that indigo molecules form a uniform monolayer on a finite number of homogeneous binding sites on the hydrogel surface. Kinetically, the system follows a pseudo-second-order model. This indicates that the overall adsorption rate is governed by chemisorption—specifically, the sharing or exchange of electrons via hydrogen bonding and hydrophobic interactions—rather than by simple physical diffusion. Thermodynamically, the process is spontaneous and endothermic. Owing to its pH sensitivity, superior swelling behavior, and magnetic responsiveness, the composite demonstrates both high adsorption efficiency and facile regeneration.

Direct dyes, such as Direct Red 80 (DR80, MW 1373 g/mol), present significant adsorption challenges due to their high molecular weight (>1000 g/mol) and bulky structures containing multiple sulfonate groups (-SO_3_^−^). Consequently, designing adsorbent architectures with enhanced swelling capabilities is critical to overcoming mass transfer limitations and steric hindrance. Addressing this, Ngwabebhoh et al. [93] constructed a chitosan-starch (ChS) semi-interpenetrating polymer network hydrogel for DR80 remediation. This architecture exhibited a substantial swelling adsorption capacity of 15 g/g, resulting in a maximum adsorption capacity of 312.77 mg/g (consistent with the Freundlich isotherm). This behavior was consistent with the Freundlich isotherm, which describes multilayer adsorption on a heterogeneous surface. This model is particularly relevant for semi-IPN hydrogels, as their diverse functional groups and complex porous domains offer binding sites with varying affinities. Kinetic analysis further revealed that, while chemisorption drives the binding, intra-particle diffusion is the rate-determining step. This means the overall adsorption rate is limited by the physical migration of the bulky DR80 molecules from the surface into the internal pores of the swollen hydrogel network, underscoring the importance of high swelling adsorption capacity for capturing large direct dyes. The hydrogel maintained high adsorption performance across four regeneration cycles, confirming its reusability. In parallel with efforts to improve sustainability and cost-efficiency, biomass waste has emerged as a promising doping agent. Grigoraș et al. [94] developed composite hydrogels by entrapping cherry stone powder within a chitosan matrix to target Acid Red 66 and Reactive Black 5. Through optimization using Response Surface Methodology (RSM), the system achieved removal efficiencies exceeding 90% in single-component solutions and 70% in binary mixtures (under optimized conditions of pH 2 and an adsorbent dosage of 100 g/L at 30 °C). These results demonstrate the effective valorization of agricultural residues in the design of functional materials.

Textile effluents typically exhibit high salinity (5–15 wt% NaCl), an environment in which traditional chitosan hydrogels exhibit uncontrolled swelling (ranging from ~300% to >1000%) and subsequent mechanical collapse (Table 2). The industrial rationale for effective dye/salt fractionation lies in the recovery of NaCl for salting-out processes and in mitigating salt inhibition during downstream biological treatment. To address this, Xie et al. [95] developed an eco-friendly hydrogel membrane Carboxymethyl chitosan-Oxalic acid-Sodium alginate (CMCS-OA-NaAlg) via the non-metallic crosslinking of sodium alginate and carboxymethyl chitosan using oxalic acid. This membrane demonstrated exceptional dimensional stability through anti-swelling behavior, maintaining a swelling ratio below 8.0% even in hypersaline conditions (10.0 wt% NaCl). Crucially, the system ultimately achieved efficient solute sieving, exhibiting dye rejection rates (e.g., Brilliant Blue, Direct Black) exceeding 95.0% while ensuring high salt permeation (NaCl rejection < 7.0%). In a parallel effort to enhance membrane permeability, Cai et al. [96] engineered a composite nano-filtration membrane by combining electrospinning technology with a chemically modified support layer. They used bacterial cellulose grafted with carboxylated multi-walled carbon nanotubes (BC-g-cMWCNTs) as a strong base, and then they covered it with a chitosan hydrogel. The resulting membrane exhibited superior mechanical integrity with a tensile strength of 11.75 MPa. Functionally, it achieved a significantly elevated water flux of 140.7 L/m^2^·h at 0.6 MPa. For dyes with molecular weights exceeding 600 g/mol, the membrane maintained rejection rates above 90%, successfully integrating high flux with robust anti-fouling properties.

The risk of secondary pollution arising from spent adsorbent disposal often plagues traditional adsorption methods for refractory dyes (Table 2). To resolve this, integrating photocatalytic functionality offers a viable pathway for in situ mineralization of captured dye molecules. Phonlakan et al. [97] constructed chitosan/Zeolitic Imidazolate Framework-8/zinc oxide (CS/ZIF-8/ZnO) composite microbeads for synergistic adsorption-photocatalytic removal of dyes, such as Reactive Red 141 (RR141). With an optimized ZIF-8 loading of 2.5%, the material exhibited a maximum adsorption capacity of 6.51 mg/g for RR141 at high concentrations (1000 mg/L). The addition of ZnO (2.59%) imparted photocatalytic properties to the composite. The system removed 99% of Methylene Blue and 90% of RR141 within 5 h when exposed to UV light. The degradation rate constants for Methylene Blue and RR141 were 0.6032 h^−1^ and 0.3198 h^−1^, respectively. The microbeads demonstrated robust stability, maintaining their performance over 10 consecutive reuse cycles.

## 5. Summary and Outlook

In recent years, substantial progress has been achieved in the remediation of antibiotics and organic dyes using chitosan-based hydrogels. A pivotal breakthrough has been the structural evolution of these materials, transitioning from conventional physical crosslinking toward more robust architectures, including chemical cross-linking, interpenetrating polymer networks, and nanocomposite systems. By rationally incorporating functional moieties, such as magnetic particles, metal–organic frameworks, and carbon nanotubes, researchers have not only significantly enhanced the mechanical integrity and chemical stability of chitosan-based hydrogels but also endowed them with advanced functionalities, including magnetic separability and photocatalytic activity. Guided by molecular-level design strategies tailored to pollutant characteristics, contemporary approaches employ aromatic functional groups to strengthen π–π stacking interactions, while electrostatic interactions are optimized through ion chelation and charge modulation. Supported by advanced characterization techniques, these studies have revealed synergistic adsorption mechanisms involving electrostatic interactions, hydrogen bonding, and coordination. As a result, engineered chitosan-based hydrogels have demonstrated exceptional performance, with reported antibiotic adsorption capacities exceeding 2000 mg/g and dye removal efficiencies consistently above 99%. Moreover, the introduction of intelligent pH- and thermo-responsive functionalities has enabled controllable desorption and excellent recyclability, with stable performance maintained over more than ten adsorption–desorption cycles. Notably, the development of integrated “adsorption–photocatalysis” systems represents a paradigm shift in remediation strategies, advancing from simple pollutant transfer to effective destructive mineralization.

Although chitosan-based hydrogels show excellent lab results for removing pollutants, applying them in real water treatment faces practical challenges. The cost and availability of raw materials, such as chitosan, can vary, affecting production costs. Manufacturing processes often involve complex steps and expensive chemicals, which may limit large-scale production. Efficient reuse of hydrogels is key to keeping costs low. Regeneration methods must keep materials effective and durable over many cycles. The materials also need to remain stable and functional over the long term across different environments. Scaling up production brings challenges in maintaining consistent quality and structure. New methods, such as continuous production or 3D printing, might help, but need more development and cost checks. To address these challenges, future research should prioritize the following directions: (1) Hierarchical Structural Regulation: Drawing inspiration from biological systems and employing advanced manufacturing techniques such as three-dimensional printing and directional freeze-casting to construct hierarchical pore architectures that synergistically enhance mass transfer efficiency and mechanical performance. (2) Intelligent and Targeted Material Design: Deep integration of artificial intelligence (AI) with materials science to develop autonomous hydrogel systems capable of “sense–decide–remediate” functions. In parallel, molecular imprinting technology (MIT) should be leveraged to improve the selective recognition and removal of trace contaminants in complex wastewater environments. (3) Holistic Degradation Platforms: Development of multifunctional “adsorption–catalysis–biodegradation” platforms to achieve full-chain pollutant mineralization. These systems should adhere to green development principles by utilizing industrial and agricultural wastes, thereby enabling a cost-effective and sustainable “waste–treat–waste” strategy. (4) Standardization and Practical Application: Establishment of standardized performance evaluation protocols and long-term environmental safety assessment frameworks is imperative. Strengthening collaboration among academia, industry, and research institutions will be essential to accelerate the translation of high-performance chitosan-based hydrogels from laboratory-scale innovation to real-world water treatment engineering applications.

## Figures and Tables

**Table 2:** Comparison of chitosan-based hydrogel adsorbents for dyes.

Material (Chitosan-Based Hydrogel/Composite)	Target (Dye Type)	Adsorption Capacity	Operating Conditions (pH, Contact Time, T)	Regeneration & Performance	Dominant Adsorption Mechanism (as Stated)	Ref.
Maleated CS grafted with acrylic & phosphonic acid	CV & MB (Cationic)	64.56 mg/g (CV); 66.89 mg/g (MB)	/	4 cycles with ethanol; stable performance	Mixed multi-mechanism (Redlich-Peterson); spontaneous & endothermic	[74]
CS gel beads embedded with SDS	CV (Cationic)	Max 76.9 mg/g	/	Extended desorption released ~47% CV, preserving SDS	Chemisorption via synergistic electrostatic (-OSO^3−^) & hydrophobic (alkyl) forces	[75]
Chitosan-coated magnetic activated carbon (Chitosan-AC-Fe_3_O_4_)	Cationic dyes	Max 505.87 mg/g	298 K	3 cycles (1 M acetic acid); desorption dropping from 64.63% to 27.84%	Multilayer physical/chemical adsorption on a heterogeneous surface (Freundlich)	[77]
Gelatin-CS magnetic hydrogel + ZnCr_2_O_4_/Fe_3_O_4_	MB & CV (Cationic)	235.5 mg/g (MB); 195.5 mg/g (CV); removal >97%	/	/	Dual “adsorption–antibacterial” synergy; metal ion slow release	[78]
CS nanocomposite hydrogels + AgNPs (RAFT synthesis)	Dyes	/	/	/	Spontaneous, endothermic; enhanced via surface polarization + antibacterial	[80]
CS/CPDA semi-IPN hydrogel (quaternized)	CR (Anionic)	Max 1803.507 mg/g	pH 4–10 stable	Stable after 6 cycles	Multilayer self-assembly on quaternary ammonium sites (Hill model)	[82]
pH-responsive magnetic CS-grafted copolymer + Fe_3_O_4_	MB (Cat.)/CR (Ani.)	1111.11 mg/g (MB); 862.06 mg/g (CR)	/	Magnetic recovery functional	Active-group enrichment with synergistic magnetic separation	[83]
CS@UiO-67 hydrogel (MOF coordinated crosslinking)	CR (Anionic)	Max 1001.2 mg/g	/	4 cycles; efficiency dropped 98% → 65%	Monolayer chemisorption: electrostatic + π-π + H-bonding	[84]
DN self-healing hydrogel (CS + oxidized SA + Ca^2+^)	MB (Cat.)/CR (Ani.)	254.41 mg/g (MB); 185.43 mg/g (CR)	/	Self-heals within 12 h	Monolayer physical/chemical interaction; robust physical recovery	[85]
Gd_2_O_3_-doped Ch-PVA-Gd hydrogel	CR (Anionic)	>99% removal; max 312.5 mg/g	pH 6; 10 min ultra-fast	/	Lewis acid sites of Gd^3+^ accelerate dye coordination	[87]
Graphene/CS aerogel	Cationic & Anionic (esp. CR)	384.62 mg/g (CR)	/	Removal near 100% over 3 cycles	π-π interactions and porous network mapping	[88]
GO/CS-PVA gel	CR (Anionic)	88.17% at pH 2; 81% at pH neutral	pH 2 optimal vs. neutral	/	Electrostatic repulsion inhibits adsorption at neutral pH (deprotonated -COOH)	[89]
CdS@n-ZnO/CS hydrogel	CR (Anionic)	95% (5mg/L); 94.34% (100mg/L)	1 min (low conc); 30 min (high conc) over sunlight	/	Adsorption + in situ photocatalytic mineralization (role of h+ and ·O^2−^)	[90]
BTCBDA-crosslinked CS + ammonium sulfate	CoTsPc (Metal complex)	98% removal	pH 8 (stable pH 4–8)	/	π-π stacking (benzophenone ↔ phthalocyanine) + ion-exchange with Co^2+^	[91]
BTDA-crosslinked CS + magnetic NPs	IC (Vat dye/Indigo)	98.9% removal	pH 4 strict optimum	Facile regeneration/responsive	Hydrophobic microdomains + H-bonding; neutral molecular state affinity	[92]
CS-starch semi-IPN hydrogel	DR80 (Direct dye)	Max 312.77 mg/g	High swelling state	High performance over 4 cycles	Multilayer chemisorption (Freundlich); intra-particle diffusion is rate-limiting	[93]
CS + cherry stone powder	Acid Red 66/Reactive Black 5	>90% (single); >70% (binary mixture)	pH 2; 30 °C	/	Enhanced matrix structure via agricultural residue embedding	[94]
CMCS-OA-NaAlg hydrogel membrane	Brilliant Blue/Direct Black	Rejection >95.0%; water permeate high	Hypersaline (up to 10.0 wt% NaCl)	/	Anti-swelling dimensional stability for dye/salt fractionation (NaCl rejection < 7%)	[95]
BC-g-cMWCNTs + CS nanofiltration membrane	Dyes (MW > 600 g/mol)	Rejection >90%; flux 140.7 L/m^2^·h	0.6 MPa pressure	Robust anti-fouling	Size sieving/nanofiltration via a hybrid support layer	[96]
CS/ZIF-8/ZnO composite microbeads	RR141/MB	6.51 mg/g (RR141 at 1000 mg/L)	5 h UV light	10 consecutive cycles robust	Synergistic adsorption + photocatalysis (RR141/MB degradation rate constant provided)	[97]

Notes: CV, Crystal Violet; MB, Methylene Blue; SDS, Sodium Dodecyl Sulfate; SA, Sodium Alginate; AC, Activated Carbon; PVA, Polyvinyl Alcohol; CPDA, Poly(dimethyldiallylammonium chloride-co-acrylamide); UiO, University of Oslo type (MOF designation); DN, Double Network; BTCBDA, 3,3′,4,4′-Benzophenonetetracarboxylic Dianhydride; BTDA, Benzophenonetetracarboxylic Dianhydride; IC, Indigo Carmine; DR80, Direct Red 80; ChS, Chitosan-Starch; CMCS, Carboxymethyl Chitosan; OA, Oxalic Acid; NaAlg, Sodium Alginate; BC-g-cMWCNTs, Bacterial Cellulose grafted with Carboxylated Multi-Walled Carbon Nanotubes; ZIF, Zeolitic Imidazolate Framework; RR141, Reactive Red 141; / Data not explicitly provided in the text. Adsorption mechanisms derived from mathematical modeling (e.g., kinetics, isotherms) are identified as proposed or inferred. In contrast, molecular interactions are considered experimentally verified only when supported by direct spectroscopic evidence (e.g., FTIR, XPS).

## Data Availability

All the data are provided in this manuscript.
